# Costs of Next-Generation Sequencing Assays in Non-Small Cell Lung Cancer: A Micro-Costing Study

**DOI:** 10.3390/curroncol29080416

**Published:** 2022-07-23

**Authors:** Srishti Kumar, Alexandria Bennett, Pearl A. Campbell, Gareth Palidwor, Bryan Lo, Theodore J. Perkins, Surapon Nochaiwong, Harmanjatinder S. Sekhon, David J. Stewart, Kednapa Thavorn

**Affiliations:** 1 Clinical Epidemiology Program, Ottawa Hospital Research Institute, Ottawa, ON K1H 8L6, Canada; srishti.kumar@evidera.com; 2 School of Epidemiology and Public Health, University of Ottawa, Ottawa, ON K1G 5Z3, Canada; d.bennett@uottawa.ca; 3 Regenerative Medicine Program, Ottawa Hospital Research Institute, Ottawa, ON K1H 8L6, Canada; pcampbell@ohri.ca (P.A.C.); gpalidwor@ohri.ca (G.P.); tperkins@ohri.ca (T.J.P.); 4 Molecular Oncology Diagnostics Laboratory, The Ottawa Hospital, Ottawa, ON K1H 8L6, Canada; brlo@toh.ca; 5 Ottawa Institute of Systems Biology, Ottawa, ON K1H 8M5, Canada; 6 Department of Biochemistry, Microbiology and Immunology, University of Ottawa, Ottawa, ON K1H 8M5, Canada; 7 Department of Pharmaceutical Care, Faculty of Pharmacy, Chiang Mai University, Chiang Mai 50200, Thailand; surapon.nochaiwong@cmu.ac.th; 8 Pharmacoepidemiology and Statistics Research Center (PESRC), Chiang Mai University, Chiang Mai 50200, Thailand; 9 Department of Pathology and Laboratory Medicine, The Ottawa Hospital, Ottawa, ON K1H 8L6, Canada; hsekhon@eorla.ca; 10 Division of Medical Oncology, The Ottawa Hospital Cancer Centre, Ottawa, ON K1H 8L6, Canada; dstewart@toh.ca

**Keywords:** next-generation sequencing, fine needle aspirates, non-small lung cancer, micro-costing

## Abstract

**Background:** Next-generation sequencing (NGS) of tumor genomes has changed and improved cancer treatment over the past few decades. It can inform clinicians on the optimal therapeutic approach in many of the solid and hematologic cancers, including non-small lung cancer (NSCLC). Our study aimed to determine the costs of NGS assays for NSCLC diagnostics. **Methods:** We performed a micro-costing study of four NGS assays (Trusight Tumor 170 Kit (Illumina), Oncomine Focus (Thermo Fisher), QIAseq Targeted DNA Custom Panel and QIASeq Targeted RNAscan Custom Panel (Qiagen), and KAPA HyperPlus/SeqCap EZ (Roche)) at the StemCore Laboratories, the Ottawa Hospital, Canada. We used a time-and-motion approach to measure personnel time and a pre-defined questionnaire to collect resource utilization. The unit costs were based on market prices. The cost data were reported in 2019 Canadian dollars. **Results:** Based on a case throughput of 500 cases per year, the per-sample cost for TruSight Tumor 170 Kit, QIASeq Targeted DNA Custom Panel and QIASeq Targeted RNAscan Custom Panel, Oncomine Focus, and HyperPlus/SeqCap EZ were CAD 1778, CAD 599, CAD 1100 and CAD 1270, respectively. The key cost drivers were library preparation (34–60%) and sequencing (31–51%), followed by data analysis (6–13%) and administrative support (2–7%). **Conclusions:** Trusight Tumor 170 Kit was the most expensive NGS assay for NSCLC diagnostics; however, an economic evaluation is required to identify the most cost-effective NGS assay. Our study results could help inform decisions to select a robust platform for NSCLC diagnostics from fine needle aspirates, and future economic evaluations of the NGS platforms to guide treatment selections for NSCLC patients.

## 1. Introduction

Recent developments in next generation sequencing (NGS) technologies have revolutionized the field of genomics by providing the ability to perform massive parallel sequencing of large areas of the genome with high accuracy [1]. The technology provides information for diagnostics, heredity risk assessment, prognosis, and treatment selection for various diseases, such as cardiovascular diseases [2], neurological diseases [3], skeletal muscle disorders [4], infectious diseases [5], and cancers [6,7]. In Canada, lung cancer accounts for 13% of new cancer cases and 25% of cancer deaths in 2022. Non-small cell lung cancer (NSCLC) is the most common type of lung cancer, with a prevalence of 80–85% of lung cancer cases [8]. The patients diagnosed with NSCLC are most often diagnosed at a late stage, as NSCLC does not show symptoms at early stages, and the estimated 5-year net survival among the late-stage NSCLC patients is as low as 0–36% [9]. Recent studies have identified several molecular drivers relevant to the initiation and progression of NSCLC; many of these mutations are actionable. The treatment guidelines for NSCLC are based on the expression of oncogenic driver gene mutations, such as EGFR and EML4-ALK. As additional drivers are identified, the complexity of clinical diagnostic test options for personalized treatment has also expanded. The use of NGS could help identify actionable gene mutations and facilitate the access to appropriate targeted therapies [10], which could improve the patient quality of life and survival outcomes [11]. However, pairing the best assay to a given sample poses a major challenge within the diagnostic laboratory, due to the limitations of diagnostic sampling, including cellular quality, quantity, and heterogeneity.

Our team at the Eastern Ontario Regional Laboratory Association and the Ottawa Hospital Research Institute conducted a research project to identify the best platform for NSCLC diagnostics, with the goal of developing a robust platform for NSCLC diagnostics from fine needle aspirates (FNAs). Although several commercial platforms exist for NSCLC-targeted diagnostics, none have undergone rigorous testing to assess their utility and associated costs in the clinical setting. The cost data are important and are relevant for resource allocation decisions at a population level. The existing studies show a substantial variation in the costs per patient of NGS testing in patients with cancer, ranging from USD 695 to USD 2861 [12,13,14,15]. The variation may be due to a difference in the methods (e.g., gross costing vs. bottom-up approach, retrospective vs. prospective design). Some of the studies calculated the total cost of the diagnostic pathway (including patient consultations and admissions), while others focused on genetic sequencing. Additionally, many of the cost studies did not fully describe the cost items included [16], and rarely used a micro-costing method [17,18], which offers a more precise assessment as it uses a direct observational approach to record the resources used along with the time it takes to fulfill a defined set of activities.

This study aimed to determine the per-sample cost of NGS assays in defining mutations in NSCLC molecular testing in Canada.

## 2. Materials and Methods

We conducted a micro-costing study and estimated per-sample costs for four NGS assays commonly used for NSCLC from a provider’s (EORLA) perspective. These assays included the Trusight Tumor 170 Kit (Illumina, San Diego, CA, USA), the Oncomine Focus (Thermo Fisher, Waltham, MS, USA), the QIAseq Targeted DNA Custom Panel and the QIAseq Targeted RNAscan Custom Panel (Qiagen, Hilden, Germany), and the SeqCap EZ/KAPA Hyper Prep Plus Custom (Roche, Basel, Switzerland). Micro-costing is a detailed costing approach in which all of the resources required for an intervention are identified and enumerated, and the unit costs are attached to these resources to estimate the total costs [17,18].

In this study, the cost of each NGS assay included the costs of library preparation, sequencing, and bio-informatic analysis and interpretation. For each of the cost components, we identified the resources consumed during each activity and divided them into the following resource use categories: (1) personnel; (2) capital; and (3) supplies and reagents. The costs were reported in 2019 Canadian dollars.

### 2.1. Data Collection

The data were collected at StemCore Laboratories at the Ottawa Hospital Research Institute over a 12-month period. We considered the resources required for the testing pathway, including capital, supplies and reagents, and personnel time to perform library preparation, sequencing, bio-informatic analysis, laboratory oversight, and training.

We used a time-and-motion approach [19,20] to measure the personnel time associated with performing the library preparation for each NGS assay. A trained research assistant tracked the time required to perform each NGS assay for a minimum of three sets of three samples to yield a test sample size of nine. A list of standard operating procedures (SOPs) was developed, based on user manuals to enumerate and define each step required to perform each assay. If the SOPs were unavailable, we conducted interviews with the clinical personnel and consultations with the clinical experts in genome sequencing to develop the detailed SOPs.

The trained research assistant recorded the data pertaining to technical hands-on time to perform each SOP, using a stopwatch. The recorded time did not include overnight incubation periods and extended periods during which the samples were on equipment with minimal-to-no staff oversight. The data on the capital required for library preparation were collected through direct observation and a pre-defined questionnaire. The data were also collected on the equipment’s lifespan, maximum capacity, and current utilization. In addition, the questionnaire was used to record information on the supplies and reagents used for each step of the SOP to perform the testing. The personnel time required for laboratory oversight and attending training was recorded, using a data collection sheet.

The data on the infrastructure required for bioinformatic analysis were collected through interviews with the bioinformatic staff, and included information on the software, storage requirements, and staff time required for data analysis, which covered time to set up an analysis pipeline for each assay, time for maintenance of an automated analysis pipeline, and time for data analysis.

The acquisition costs for capital and the unit costs for supplies and reagents were based on market prices. The unit costs for supplies and reagents were derived by dividing their market price by the number of units contained within the item. Staff salaries were obtained from the budget reviews and were verified by the team. The midpoint of the salary ranges was used, and a working week was assumed to be 37.5 h and a working year was assumed to be 52 weeks. The costs of performing sequencing were obtained as lump-sum costs from the study team, and included the costs of required equipment and personnel time to perform sequencing.

### 2.2. Analysis

The cost for each assay was equal to a sum of the costs of personnel, equipment and consumables across library preparation, sequencing, bioinformatic analysis, and administrative support. We calculated the total costs as a product of the unit costs and resource use. We estimated the costs for DNA and RNA samples, except for SeqCap EZ/KAPA Hyper Prep Plus Custom in which only the cost for the DNA samples was reported, as the assay was not compatible with RNA.

Guided by expert opinion, we assumed that the capital required for library preparation is comparable across the assays. For the costs of equipment, the acquisition cost for each unit was amortized over the lifespan of the unit at a discount rate of 1.5% per annum [21]. The personnel costs for library preparation, bioinformatic analysis, administrative support, and training were estimated as a product of the time required to perform an activity and mean wage estimates. For the personnel costs related to administrative support and training, the annual costs were estimated based on the attributable full-time equivalent in a year. The costs for supplies and reagents for each phase were estimated by multiplying the number of units required for an activity by the unit costs based on market prices.

The costs for each assay were also categorized into fixed and variable costs. The fixed costs are those that do not vary by case throughput (e.g., capital), while the variable costs are defined as costs that vary by case throughput (e.g., personnel costs for library costs and analysis). The per-sample cost for each assay was estimated by dividing the annualized costs by the annual laboratory throughput, which was based on the annual laboratory case throughput at the StemCore Laboratories. We performed a one-way sensitivity analysis to assess the uncertainty in the cost estimates by varying the annual case throughput and batch size between their respective upper and lower bounds, which were determined based on clinical expert opinion.

## 3. Results

The per-sample cost to process a DNA sample was CAD 1287.87 for the Trusight Tumor 170 Kit, CAD 1227.93 for the SeqCap EZ/KAPA Hyper Prep Plus Custom, CAD 1005.33 for the Oncomine Focus, and CAD 449.32 for the QIAseq Targeted DNA Custom Panel (Table 1). The per-sample cost to process an RNA sample was CAD 1245.95 for the Trusight Tumor 170 Kit, CAD 1001.75 for the Oncomine Focus, and CAD 586.70 for the QIASeq Targeted RNAscan Custom Panel (Table 1). The Trusight Tumor 170 Kit was the most expensive assay for both the DNA and RNA samples. The library preparation (38–62%) and sequencing (32–54%) were the main drivers of the assay costs, followed by administrative support (3–8%), and bio-informatic analysis (1–3%) (Figure S1).

Table 2 presents the annual capital costs for each assay by testing steps. The differences in the capital costs across the assays were driven by the costs of software required for the bioinformatic analysis. The annual capital cost was highest for the Targeted DNA and RNA Panels (CAD 31,142).

Table 3 presents a breakdown of the per-sample variable costs for the DNA and RNA samples for each assay by testing steps. The differences in the variable costs across the assays were driven by the costs of the library preparation kit and sequencing activity. The per-sample costs for the DNA and RNA samples were comparable for the Oncomine Focus and the SeqCap EZ/KAPA Hyper Prep Plus Custom. For the Trusight Tumor 170 Kit and the QIAseq Targeted DNA and RNAscan Custom Panels, the differences in the costs between the DNA and RNA samples were observed in the costs of consumables and personnel time for library preparation and sequencing.

## 4. Discussion

Our study used a micro-costing approach to estimate the costs of four high-throughput genomic assays in NSCLC. The costs accounted for the personnel time, capital, and the supplies and reagents required for library preparation, sequencing, and bioinformatic analysis. Our study showed that the per-sample cost was highest for the Trusight Tumor 170 Kit (DNA: CAD 1288; RNA: CAD 1246), followed by the SeqCap EZ/KAPA Hyper Prep Plus Custom (DNA: CAD 1228), the Oncomine Focus (DNA: CAD 1005; RNA: CAD 1002), and the QIAseq Targeted DNA and RNAscan Custom Panels (DNA: CAD 471; RNA: CAD 609). It should be noted that each assay may have different performance characteristics and that the Trusight Tumor 170 Kit is the largest panel, which covers 170 tumor-related genes, meaning that the assay would allow the sequencing of many tumor genes, including those not yet approved for NSCLC diagnostics, without additional costs. However, the assay’s performance is beyond the scope of this study. Regardless of the type of assay, the NGS costs were primarily driven by the supply and reagent costs for library preparation (23–51%) and sequencing (32–52%). The fixed costs of capital accounted for only 5–10% of the total costs. These cost estimates were robust to changes in the case throughput and batch size (Table 4).

### Comparison with Literature

The costs of the NGS assays estimated in our study were within the range of costs reported in previous cost studies, which evaluated the cost of genomic sequencing procedures in NSCLC patients. Sabatini et al. [22] used a micro-costing approach to estimate the cost of genomic sequencing procedures for: (i) patients with advanced NSCLC who require treatment optimization; (ii) patients being evaluated for syndromic sensorineural hearing loss; and (iii) children experiencing neurodevelopmental disorders. The authors considered the development, validation, maintenance, quality control, and overhead costs and reported that the costs of targeted genomic sequence analysis of DNA from solid tumor specimens ranged from USD 577.99 to USD 907.82 (CAD 816–CAD 1281), and for a tumor panel with >50 genes was USD 1948 (CAD 27,489). Consistent with our study, the key cost drivers were the consumables for library preparation and sequencing, bioinformatics, and overhead costs. Similarly, a Dutch study [23] used an activity-based costing approach to estimate the costs for NGS panels (small- and medium-targeted gene panel (TGP)) for patients with stage IV NSCLC and melanoma, and showed that the costs varied by batch size, number of runs, and type of TGP. The per-sample costs for the small TGP ranged between EUR 606–EUR 956 (CAD 1441–CAD 2273), and that for the medium TGP was between EUR 1137–EUR 3009 (CAD 2703–CAD 7154). Moreover, a study by Johnston et al. [24] characterized the costs of conventional in-house diagnostic testing for NSCLC in Canada, using data collected from structured interviews with oncologists, pathologists, and laboratory directors. The study included the upfront costs of equipment and initial employee training and the operational expenses of equipment maintenance, consumables, and personnel time. The total cost was CAD 652 per single gene and CAD 1919 per panel. Total test material costs were CAD 133 per single-gene test and CAD 1400 per panel, and infrastructure costs were an additional CAD 518.75 per test.

Our study had some limitations that must be acknowledged. First, the data were collected from a single setting; our results may therefore not be generalizable to other settings, as the personnel time required to process a sample could vary across laboratories. The costs could change once the assays are moved into clinical practice and become part of the provincially funded system, due to additional costs relating to quality assurance protocols, expanded bio-informatics support, management, and oversight [1]. Second, the unit costs for equipment and consumables were based on the market prices and are subject to variation over time and by setting. Lastly, our study applied a direct observation technique to collect data on personnel time for library preparation for each assay. This approach may be subject to the Hawthorne effect, which is the tendency for the performance of an activity to be altered to seem more favorable to the observer [20]. Our study attempted to mitigate this effect through the collection of multiple observations, verification of estimates by laboratory personnel, and external validation using the results from existing literature. Despite these limitations, our study contributes to the existing evidence by presenting comprehensive cost estimates for NGS assays for NSCLC samples in Canada. The transparency of a micro-costing approach and the results allow future studies to update the costs as the technology evolves [25]. Based on our costing results, researchers could perform a cost-effectiveness analysis of commercially available platforms by comparing their costs and outcomes with respect to sensitivity, specificity, limits of detection, reproducibility, comprehensiveness, and turnaround time. Such an analysis could help select the optimal assay(s) for NSCLC FNAs in the clinical setting. Future studies should assess the impact of sequencing at scale on the NGS costs. The results from our study could also be used to inform future economic evaluations and budgetary impact calculation of genomic sequencing and targeted therapies for NSCLC. Furthermore, understanding the key drivers of the costs for NGS assays could inform the decisions on the implementation of these assays to the publicly funded health system.

## Figures and Tables

**Table 1:** Per-sample costs by NGS assays and testing steps.

	Library Preparation (CAD)	Sequencing (CAD)	Bio-Informatic Analysis (CAD)	Administrative Support and Training (CAD)	Total (CAD)
DNA					
Trusight Tumor 170 Kit	743.11	496.25	10.07	38.43	1287.87
Targeted DNA Panels	244.38	184.80	3.75	38.43	471.36
Oncomine Focus	452.08	504.75	10.07	38.43	1005.33
SeqCap EZ/KAPA Hyper Prep Plus Custom	756.24	393.17	40.09	38.43	1227.93
RNA					
Trusight Tumor 170 Kit	701.19	496.25	10.07	38.43	1245.95
QIAseq Targeted RNAscan Custom Panels	196.97	369.60	3.75	38.43	608.74
Oncomine Focus	448.50	504.75	10.07	38.43	1001.75

**Table 2:** Annual fixed costs of NGS, by assay.

Testing Step	Subcomponent	Trusight Tumor 170 Kit (CAD)	QIAseq Targeted DNA and RNAscan Custom Panels (CAD)	Oncomine Focus (CAD)	SeqCap EZ/KAPA Hyper Prep Plus Custom (CAD)
Library Preparation	Equipment	23,596.84	23,596.84	23,596.84	23,596.84
Bioinformatic Analysis	Software for bioinformatic analysis and fixed costs of personnel time to set up and maintain an automated analysis pipeline *	4836.36	1672.71	4836.36	7345.43
	Data storage	200	200	200	200
Annual Fixed Costs		28,633.20	25,469.56	28,633.20	31,142.27

* For Trusight Tumor 170 Kit, Oncomine Focus, and SeqCap EZ/KAPA Hyper Prep Plus Custom, the costs indicate amortized costs of personnel time to set up an automated analysis pipeline and the yearly personnel costs to maintain it. For QIAseq Targeted DNA and RNAscan Custom Panels, the value indicates the amortized acquisition cost of software.

**Table 3:** Variable costs per sample, by assay.

Testing Step	Subcomponent	Trusight Tumor 170 Kit (CAD)	QIAseq Targeted DNA and RNAscan Custom Panels (CAD)	Oncomine Focus (CAD)	SeqCap EZ/KAPA Hyper Prep Plus Custom (CAD)
DNA					
Library Preparation	Supplies and reagents	48.57	48.57	48.57	48.57
	Library preparation kit	564.45	126.37	327.54	625.22
	Personnel	82.91	22.24	28.77	35.26
Sequencing *		496.25	184.80	504.75	393.17
Bio-informatic Analysis	Personnel	0.00	0.00	0.00	0.00
Administrative Support	Administrative support	36.16	36.16	36.16	36.16
	Training	2.27	2.27	2.27	2.27
Total Variable Cost, Per Sample		1230.60	420.42	948.06	1165.64
RNA					
Library Preparation	Supplies and reagents	48.57	48.57	48.57	NA
	Library preparation kit	564.45	73.79	323.96	NA
	Personnel	40.98	27.41	28.77	NA
Sequencing *		496.25	369.60	504.75	NA
Bio-informatic Analysis	Personnel	0.00	0.00	0.00	NA
Administrative Support	Administrative support	36.16	36.16	36.16	NA
	Training	2.27	2.27	2.27	NA
Total Variable Cost, Per Sample		1188.68	557.80	944.48	NA

* All sequencing costs were assumed to be variable; NA, not available.

**Table 4:** One-way sensitivity analyses: per-sample costs by annual case throughput and batch size.

Parameter	Trusight Tumor 170 Kit (CAD)		QIAseq Targeted DNA and RNAscan Custom Panels (CAD)		Oncomine Focus (CAD)		SeqCap EZ/KAPA Hyper Prep Plus Custom (CAD)	
	LB *	UB *	LB *	UB *	LB *	UB *	LB *	UB *
Annual Case Throughput ** LB = 250; UB = 750	1362.60	1235.01	610.20	516.67	1099.24	971.64	1328.64	1194.35
Batch size *** LB = 3; UB = 8	1291.79	1266.91	558.90	540.05	1007.46	1003.54	1254.07	1227.93

* LB = Lower bound for parameter varied in sensitivity analysis; UB = Upper bound for parameter varied in sensitivity analysis; ** units = cases per years; *** units = cases per run.

## Data Availability

The data that support the findings of this study are available from the corresponding author, (K.T.), upon reasonable request.

## Supplementary Materials

The following are available online at https://www.mdpi.com/article/10.3390/curroncol29080416/s1, Figure S1: Distribution of per-sample cost by testing step.
