# Supplementary material for: Costs of Next-Generation Sequencing Assays in Non-Small Cell Lung Cancer: A Micro-Costing Study
Source: Curr Oncol. 2022 Jul 23;29(8):5238–46. doi: 10.3390/curroncol29080416 (PMC9330154; doi:10.3390/curroncol29080416)
Supplement: Supplementary file 1 [file curroncol-29-00416-s001.zip › curroncol-1770611-supplementary.pdf]

a.

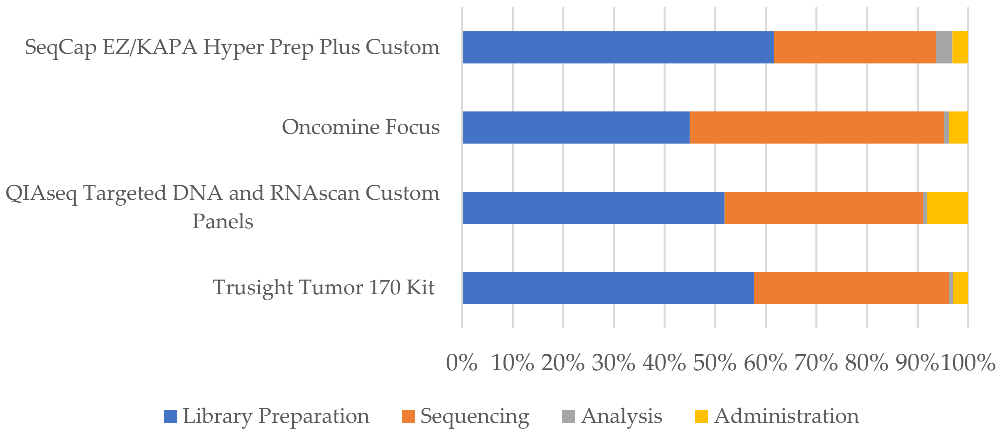

b

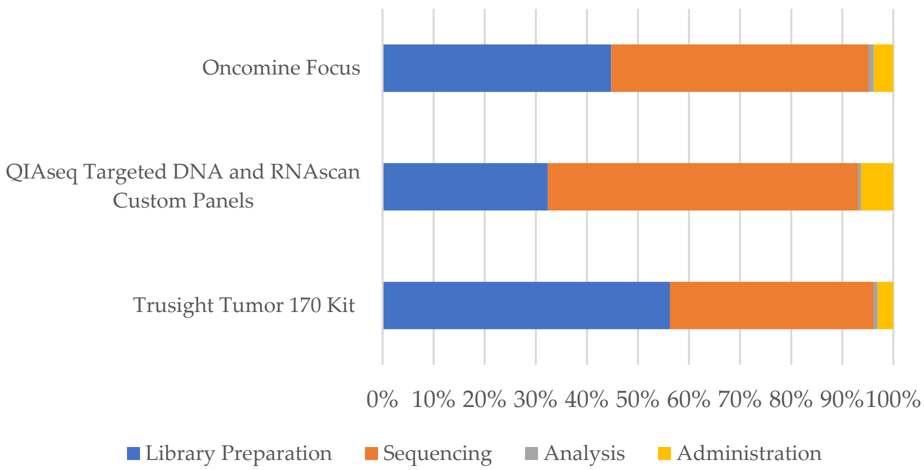

Figure S1. Distribution of per-sample cost by testing step. (a)This figure shows the distribution of per-sample costs by testing step in DNA samples; (b) Distribution of per-sample cost by testing step, RNA samples.
